# Blood N‐glycomics reveals individuals at risk for cognitive decline and Alzheimer’s disease

**DOI:** 10.1002/alz70861_108302

**Published:** 2025-12-23

**Authors:** Robin Ziyue Zhou, Stefan Gaunitz, Anders Skoldunger, Anders Wimo, Bengt Winblad, Tormod Fladby, Sophia Schedin Weiss, Lars O Tjernberg

**Affiliations:** ^1^ Karolinska Institutet, Stockholm, Stockholm Sweden; ^2^ Karolinska University Hospital, Huddinge, Stockholm Sweden; ^3^ University of Oslo, Oslo Norway; ^4^ Karolinska Institutet, Solna, Stockholm Sweden

## Abstract

**Background:**

It is increasingly clear that aberrant N‐glycosylation of proteins is an early pathogenic trigger in Alzheimer’s disease (AD). Thus, we hypothesized that determination of the blood N‐glycome could reveal AD‐specific glycomic patterns, especially in early stages of the disease.

**Method:**

For N‐glycan quantification in blood, we developed a method based on porous graphite carbon liquid chromatography coupled to tandem mass spectrometry (PGC‐LC‐MS/MS). We simultaneously determined levels of 62 N‐glycan structures in 100 individuals from two different cohorts. In the Swedish National Study on Aging and Care in Nordanstig (SNAC‐N) cohort, we analyzed blood samples from 40 age‐ and sex‐matched AD and control individuals. In the Norwegian Dementia Disease Initiation (DDI) cohort, we analyzed blood samples from 60 individuals from A‐T‐, A+T‐, and A+T+ biomarker groups, matched by age, sex, and education.

**Result:**

In the Swedish SNAC‐N discovery cohort, a specific blood signature of low levels of a group of N‐glycans was associated with AD dementia, lower cognitive function, as well as higher levels of AD biomarkers such as blood pTau217. In the Norwegian DDI cohort, a similar low N‐glycosylation blood signature was associated with *APOE4* genotype and future cognitive decline, but not with A/T status. In short, low levels of 14 N‐glycan structures were associated with poor cognitive outcome in both cohorts.

**Conclusion:**

Here, we provided an extensive characterization of the blood N‐glycome in AD. We showed that the blood N‐glycome could predict cognitive decline in an amyloid/tau independent manner. Blood N‐glycomics should be further developed as a minimally invasive prognostic biomarker in early stages of dementia.

